# Are Behavioral Tests Capable of Measuring Positive Affective States in Growing Pigs?

**DOI:** 10.3390/ani9050274

**Published:** 2019-05-24

**Authors:** Katja Krugmann, Farina Warnken, Joachim Krieter, Irena Czycholl

**Affiliations:** Institute of Animal Breeding and Husbandry, Christian-Albrechts-University Kiel, Olshausenstr. 40, D-24098 Kiel, Germany; fwarnken@tierzucht.uni-kiel.de (F.W.); jkrieter@tierzucht.uni-kiel.de (J.K.); iczycholl@tierzucht.uni-kiel.de (I.C.)

**Keywords:** animal welfare, human approach test, novel object test, positive emotions, pigs

## Abstract

**Simple Summary:**

This study aimed at deriving potential indicators to assess fattening pigs’ positive affective state in order to be able to assess animal welfare more objectively. For this purpose, 297 fattening pigs from two different housing systems (a barren and an enriched environment) were subjected three times to the human approach test and novel object test (at the start, middle and end of fattening). The barren-housed pigs showed quicker approach latencies to come into contact with the unknown human and the novel object compared to the enriched-housed pigs (e.g., latency time in the human approach test at the end of fattening: barren housing system: 7.4 ± 1.1 s vs. enriched housing system: 57.1 ± 1.1 s, respectively 58.3 ± 1.3 s). They also indicated longer durations of contact in the human approach test but not in the novel object test (e.g., duration of contact in the human approach test at the end of fattening: barren housing system: 83.8 ± 1.1 s vs. enriched housing system: 6.3 ± 1.1 s respectively, 7.6 ± 1.3 s). However, taking the literature into account, interpretation of these results is not straightforward as the reasoning for these findings could be boredom, and thus a higher motivation to explore, or less fear. Hence, behavioral tests as solitary indicators are probably less useful in the assessment of an affective state.

**Abstract:**

This study examined whether the human approach test (HAT) or novel object test (NOT), which are considered as suitable tests for assessing the level of fear or anxiety in animals, are suitable to detect a positive affective state in 297 fattening pigs from three different farms. The investigated farms consisted of a barren (farm 1, *n* = 160) and an enriched (farm 2, *n* = 106; farm 3, *n* = 31) husbandry. Each pig was subjected three times to the HAT and NOT during fattening (at the start, middle, and end of fattening). The pigs housed in the barren environment showed quicker approach latencies than the enriched-housed pigs (HAT: farm 1: 7.4 ± 1.1 s vs. farm 2: 57.1 ± 1.1 s, respectively, farm 3: 58.3 ± 1.3 s (end of fattening); NOT: farm 1: 4.5 ± 1.1 s vs. farm 2: 23.0 ± 1.1 s, respectively, farm 3: 9.0 ± 1.2 s (end of fattening)). The same pattern of behavior was observed for the duration of contacts in the HAT but not in the NOT (HAT: farm 1: 83.8 ± 1.1 s vs. farm 2: 6.3 ± 1.1 s, respectively, farm 3: 7.6 ± 1.3 s (end of fattening)). However, due to controversially discussed literature, it is difficult to conclude whether the described differences in the pigs’ behavior between the two housing systems might indicate useful indicators to detect their affective state.

## 1. Introduction

Certain behavioral reactions of animals are commonly measured by behavioral tests performed in standardized environments and seem to capture some parts of the behavioral tendencies of the individual animal [1]. Thus, implementations of behavioral tests are also used in breeding decisions [2]. A large number of behavioral tests focus on assessing emotions [3], whereas the human approach test (HAT) and novel object test (NOT) are known to be suitable to measure the level of fear or anxiety in animals [4,5]. Moreover, these tests are also used to evaluate the effectiveness of medical treatments such as anxiolytics [6]. Hence, the reactions to sudden or unfamiliar events are used to assess the animals’ fearfulness [7], whereby the amount of avoidance or approach behavior is suggested to provide a measure of the level of fear in animals [5]. Accordingly, a shorter latency to approach novel stimuli is maintained to show less fearful animals [1], so that it could be imagined that these animals also possess a more positive affective state since fear is detected as a strong negative emotion, which is also often included in assessments of animal welfare [1]. The affective state, which is determined as an essential part of animal welfare, constitutes the experienced positive and negative emotions of animals [8]. However, it still poses an enormous challenge to measure the affective state of animals objectively as previous assessment systems are partially unreliable and strongly influenced by subjective perceptions [9,10,11,12]. The generally criticized intensive housing conditions of farm animals and a greater demand for more “animal friendly” systems, and the necessity to detect reliable and suitable indicators that identify animals’ especially positive affective state [13] have all continued to gain significance in the course of the increasing interest in animal welfare.

It is conceivable that behavioral features to assess fear [3], such as the latency to approach and contact novel stimuli, but also other measures, such as the duration or number of contacts, provide valuable information on behavioral patterns of animals. Aside from measuring the level of fear, the behavioral variables mentioned reflect the willingness to explore novel objects, although the boldness of the animals’ personality can also be measured through responses to novel objects, threatening stimuli, or environments [14]. Furthermore, van der Staay et al. [15] suggest that the affective state of animals is influenced by environmental conditions. Moreover, primarily the NOT appears to be sensitive to marginal changes in the environment [16]. Hence, it could be imaginable that animals which are housed in differing environments (e.g., in terms of barren or enriched habitats, different handling treatments, or further stimuli), react in different ways to sudden or unfamiliar events as are said to be measured in behavioral tests [7]. These expected different behavioral reactions could be useful to obtain a better comprehension of the affective state of farm animals.

Thus, the present study aimed at determining behavioral patterns of fattening pigs housed in different habitats that could be measured in the HAT and the NOT to detect suitable behavioral indicators to assess a livestock’s affective state. It has been hypothesized that particularly quicker approach latency (AL) and longer duration of contact (DC) indicate less fearful and more curious pigs that might simultaneously possess a more positive affective state.

## 2. Materials and Methods

### 2.1. Animals and Housing Conditions

The behavioral data were collected on three farms with different housing systems in Northern Germany between November 2016 and September 2017. A total of 297 fattening pigs bred from commercial crossbred dams (Large White × Landrace) and sired by Pietrain boars were tested in total. The tails of the pigs were undocked, and the boars were castrated. The housing systems differed primarily in terms of the space availability (m^2^/pig), whether it was an enriched or barren habitat, and the climatic conditions.

Farm 1 was a fattening stable in which the pigs were kept in groups of 19 to a pen. The pens measured 3.70 × 4.70 m resulting in 0.92 m^2^ per pig. The floor was half planed, and half perforated with no bedding. These pigs were fed ad libitum with pelleted feed through a dry-feeding machine and water was accessible through nipple drinkers. The ambient temperature was 18 °C. There was daily artificial light for eight hours (07:00–15:00).

An ecological fattening stable which included inside and outside pens (in total 4.90 × 9.80 m) with straw bedding represented Farm 2. There was a roofed (2.70 × 4.90 m) and roofless area (3.50 × 4.90 m) in the outside pens. At the beginning of fattening, 54 pigs were housed in one pen with a total area of 48.02m^2^ (4.90 × 9.80 m) resulting in 0.89 m^2^ per pig. Throughout fattening the animals were divided into three of these pens evenly so that each pig attained a space of 2.67 m^2^. These pigs were fed ad libitum with mealy feed.

Farm 3 constituted an ecological fattening stable with straw-bedded inside pens and roofed straw-bedded outside pens, as well as outside pens with soiled floor for rooting. The dimension of these pens measured in total 83.32 m^2^ (8.33 m^2^ per pig with a usual occupancy of 10 pigs per pen). The feeding was ad libitum with liquid feed. 

On Farm 2 and Farm 3 there were also nipple drinkers and hayracks available and the daylight length as well as the ambient temperature were determined by the season. Once a day these pigs got bread and different fruits and vegetables.

Farm 1 was considered as a barren housing environment due to the half-planed and half-perforated floor without bedding. The straw-bedded inside and outside pens, respectively, soil-based rooting areas, hayracks, and different fruits and vegetables were determined as enrichment of the housing environment of Farm 2 and Farm 3.

Husbandry practices on all farms were similar and included daily routine animal control (i.e., visual inspections of the pigs, the feeding and drinking systems, and further pen equipment). Human–animal interactions were reduced as much as possible.

### 2.2. Experimental Procedure

A total of 297 fattening pigs of three different farms were subjected to three HATs and three NOTs during their fattening. The first, second, and third points of testing were conducted at the beginning, middle, and end of fattening. In each batch the same pigs were tested at each point of testing. Each pig was tested alone in the home pen. Both behavioral tests were performed with a one-day time lag in between and never on the same day. The pigs were given an acclimation period of two minutes followed by a test period of three minutes that started when the unknown human entered, or the novel object were brought into the test area. The unknown human in the HAT was always a female person the pigs did not know from daily routine work. She wore a clean overall plus rubber boots and stood motionless in the middle of the pen during the entire test phase. A plastic duck presented to the pigs in three different sizes (according to the age and the live weight of the animals) represented the utilized novel objects in the NOT. These ducks showed a yellow body color with a red colored beak. With the help of a rod and a string, they were held in the middle of the pen at the height of the pig’s head. Always the same observing person noted the analyzed variables during the test phase: the approach latency (AL), the duration of contact (DC), and the number of contacts (NC). AL represented the time in seconds that each pig needed to approach the unknown human or the novel object until the snout touched the human or the novel object. DC exposed the accumulated seconds in which the pigs touched the human or the novel object with their snouts. The entire number of snout contacts that occurred during the test phase was indicated by NC. Further, an AL of 180 s was noted if the pigs did not come into contact with the human or the novel object.

The behavioral tests were conducted in the home pens of the respective housing systems. On Farm 1 the home pen (3.70 × 4.70 m) was used completely. Parts of the outdoor area were used for testing the pigs on the other two farms. The amount of space for testing on Farm 2 measured 6.20 × 4.90 m with a roofed and unroofed area. On Farm 3, the roofed outdoor area was utilized (2.40 × 3.80 m).

### 2.3. Statistical Analysis

Statistical analyses were performed with SAS^®^ 9.4 [17]. The behavioral data were not normally distributed. Thus, all data were log10 (X + 1) transformed to obtain normality of residuals of the used linear mixed model (PROC MIXED). Fixed effects were added to the model in a stepwise manner. The Akaike’s information criteria corrected (AICC) and the Bayesian information criteria (BIC) were used to compare the different models. The model with the smallest AICC and BIC values was chosen and included the fixed effects farm (1–3), batch (1,2) nested in farm, points of testing (beginning, middle and end of fattening) nested in farm, and gender (female, male) together with a random effect of each individual pig nested in farm, batch, and gender. The significance of differences in the least square means was adjusted with the Bonferroni correction. Statistical significance was determined at *p* < 0.05.

The residuals of the linear mixed models of all behavioral variables of both behavioral tests were correlated through the Pearson correlation coefficient (PROC CORR).

## 3. Results

### 3.1. Human Approach Test (HAT)

#### 3.1.1. Approach Latency (AL) HAT

Differences Between the Farms at Each Point of Testing

**Figure 1:**
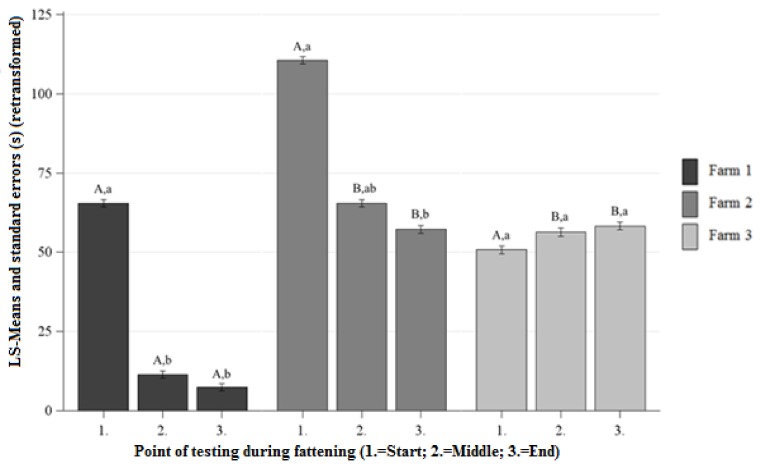
Least squares means-means and standard errors (in seconds) (retransformed) of the approach latency in the human approach test (HAT) at each point of testing; ^a, b, c^ letters indicate significant differences within the farm between the different points of testing (*p* < 0.05); ^A, B, C^ letters indicate significant differences between the farms within the different points of testing (*p* < 0.05).

No significant differences between the AL were observed on Farm 1 and Farm 2 at the beginning of fattening (Figure 1). On Farm 1, the AL was significantly lower than on Farm 2 at the middle and the ending of the fattening (Figure 1). There were no significant differences between the AL of Farm 1 and Farm 3 at the beginning of the fattening, and significantly lower AL on Farm 1 than on Farm 3 at the middle and the ending of the fattening (Figure 1). No significant differences between the AL were observed on Farm 2 and Farm 3 at the beginning, middle, and ending of the fattening (Figure 1).

Differences Between the Points of Testing Within Each Farm

The AL significantly decreased throughout fattening from the beginning to end on Farm 1 and Farm 2 (Figure 1). On Farm 3, no significant differences in the AL were observed between the beginning and end of fattening (Figure 1).

#### 3.1.2. Duration of Contact (DC) HAT

##### Differences Between the Farms at Each Point of Testing

**Figure 2:**
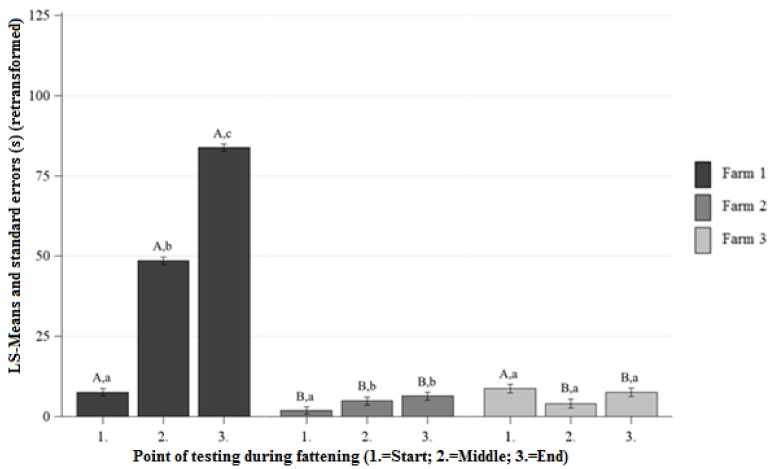
Least squares means-means and standard errors (in seconds) (retransformed) of the duration of contact in the HAT at each point of testing; ^a, b, c^ letters indicate significant differences within the farm between the different points of testing (*p* < 0.05); ^A, B, C^ letters indicate significant differences between the farms within the different points of testing (*p* < 0.05).

At the beginning, middle, and end of fattening the pigs on Farm 1 showed longer DC than the pigs on Farm 2 (Figure 2). There were no significant differences observed between the DC of Farm 1 and Farm 3 at the beginning of fattening (Figure 2). At the middle and end of fattening, the pigs on Farm 1 showed longer DC than the pigs on Farm 3 (Figure 2). On Farm 2, the DC was significantly lower than on Farm 3 at the beginning of fattening and there were no significant differences between the DC on Farm 2 and Farm 3 at the middle and end of fattening (Figure 2).

##### Differences Between the Points of Testing Within Each Farm

On Farm 1 and Farm 2, the DC significantly increased from the beginning to the end of fattening (Figure 2). On Farm 3, there was no significant increase or decrease of the DC between the beginning and end of fattening observed (Figure 2).

#### 3.1.3. Number of Contacts (NC) HAT

##### Differences Between the Farms at Each Point of Testing

At the beginning and middle of fattening the pigs on Farm 1 showed significantly higher NC than the pigs on Farm 2 (beginning of the fattening: farm 1: 1.8 ± 1.0 s vs. farm 2 1.3 ± 1.0 s; *p* < 0.05; middle of the fattening: farm 1: 2.9 ± 1.0 s vs. farm 2: 1.8 ± 1.0 s; *p* < 0.05). At the end of fattening there were no significant differences between the NC of Farm 1 and Farm 2 (farm 1: 2.3 ± 1.0 s vs. Farm 2: 1.9 ± 1.0 s; *p* > 0.05). There were no significant differences observed between the NC of Farm 1 and Farm 3 at the beginning and end of fattening (beginning of fattening: Farm 1: 1.8 ± 1.0 s vs. Farm 3: 1.7 ± 1.1 s; *p* > 0.05; end of fattening: Farm 1: 2.3 ± 1.0 s vs. Farm 3: 1.8 ± 1.1 s; *p* > 0.05). At the middle of fattening, the pigs on Farm 1 showed significantly higher NC than the pigs on Farm 3 (Farm 1: 2.9 ± 1.0 s vs. Farm 3: 1.5 ± 1.1 s; *p* < 0.05). On Farm 2, there was significantly lower NC than on Farm 3 at the beginning of fattening (Farm 2: 1.3 ± 1.0 s vs. Farm 3 1.7 ± 1.1 s; *p* < 0.05) and at the middle and end of fattening there were no significant differences between the NC of Farm 2 and Farm 3 (middle of fattening: Farm 2: 1.8 ± 1.0 s vs. Farm 3 1.5 ± 1.1 s; *p* > 0.05); end of fattening: Farm 2: 1.9 ± 1.0 s vs. Farm 3: 1.8 ± 1.1 s; *p* > 0.05).

##### Differences Between the Points of Testing Within Each Farm

There was a significant increase in NC on Farm 1 and Farm 2 from the beginning to the end of fattening (Farm 1: 1.8 ± 1.0 s vs. 2.3 ± 1.0 s; *p* < 0.05); Farm 2: 1.3 ± 1.0 s vs. 1.9 ± 1.0 s; *p* < 0.05) and no significant differences between the NC of the beginning and end of fattening on Farm 3 (1.7 ± 1.1 s vs. 1.8 ± 1.1 s; *p* > 0.05).

#### 3.1.4. Gender HAT

The female pigs showed significantly lower AL and longer DC than the castrated males (Table 1). No significant differences were observed between the NC of the female and castrated male pigs (Table 1).

### 3.2. Novel Object Test (NOT)

#### 3.2.1. Approach Latency (AL) NOT

##### Differences between the Farms at Each Point of Testing

**Figure 3:**
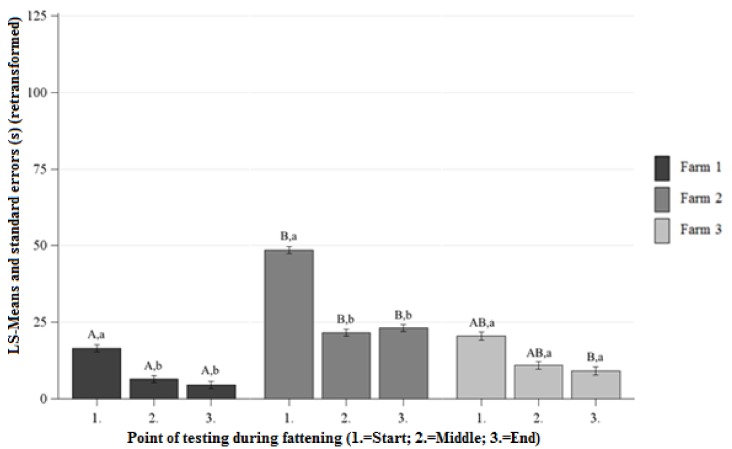
Least squaresmeans and standard errors (in seconds) (retransformed) of the approach latency in the NOT at each point of testing; ^a, b, c^ letters indicate significant differences within the farm between the different points of testing (*p* < 0.05); ^A, B, C^ letters indicate significant differences between the farms within the different points of testing (*p* < 0.05).

At the beginning, middle, and end of fattening significantly lower AL was observed on Farm 1 than on Farm 2 (Figure 3). At the beginning and middle of fattening there were no significant differences between the AL on Farm 1 and Farm 3. At the end of fattening the pigs on Farm 1 showed lower AL than the pigs on Farm 3 (Figure 3). There were no significant differences between the AL on Farm 2 and Farm 3 at the beginning, middle, and end of fattening (Figure 3).

##### Differences between the Points of Testing Within Each Farm

The AL on Farm 1 and Farm 2 decreased from the beginning to the end of fattening (Figure 3). No significant differences of the AL were observed at the beginning and end of fattening on Farm 3 (Figure 3).

#### 3.2.2. Duration of Contact (DC) NOT

##### Differences Between the Farms at Each Point of Testing

**Figure 4:**
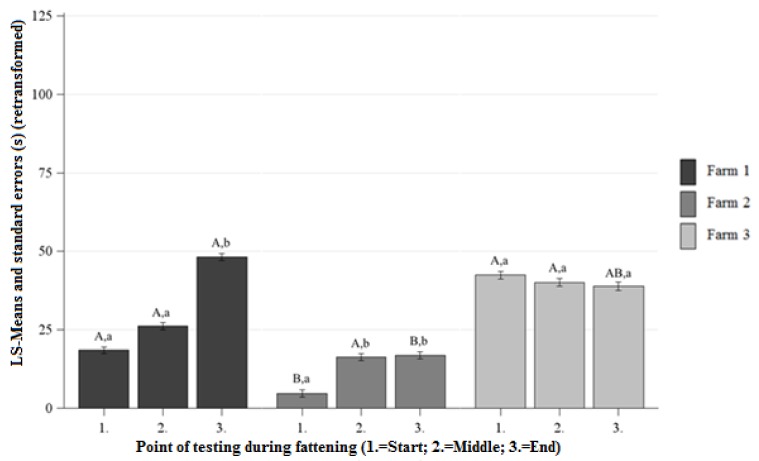
Least squaresmeans and standard errors (in seconds) (retransformed) of the duration of contact in the NOT at each point of testing; ^a, b, c^ indicate significant differences within the farm between the different points of testing (*p* < 0.05); ^A, B, C^ letters indicate significant differences between the farms within the different points of testing (*p* < 0.05).

There was longer DC on Farm 1 than on Farm 2 at the beginning and end of fattening and no significant differences between the DC of Farm 1 and Farm 2 at the middle of fattening (Figure 4). On Farm 1 and Farm 3 there were no significant differences observed between the DC at the beginning, middle, and end of fattening (Figure 4). At the beginning of fattening the pigs on Farm 2 showed shorter DC than the pigs on Farm 3 and there were no significant differences between the DC of Farm 2 and Farm 3 at the middle and end of fattening (Figure 4).

##### Differences Between the Points of Testing Within Each Farm

The pigs on Farm 1 and Farm 2 showed a significant increase in DC from the beginning to the end of fattening and there were no significant differences between the DC of the beginning and end of fattening on Farm 3 (Figure 4).

#### 3.2.3. Number of Contacts (NC) NOT

##### Differences Between the Farms at Each Point of Testing

At the beginning, middle, and end of fattening the pigs on Farm 1 showed significantly higher NC than the pigs on Farm 2 (beginning of fattening: Farm 1: 3.3 ± 1.0 s vs. Farm 2 1.9 ± 1.0 s; *p* < 0.05; middle of fattening: Farm 1: 3.6 ± 1.0 s vs. Farm 2: 2.8 ± 1.0 s; *p* < 0.05; end of fattening: Farm 1: 4.3 ± 1.0 s vs. Farm 2: 2.5 ± 1.0 s; *p* < 0.05). At the beginning, middle, and end of fattening there were no significant differences between the NC of Farm 1 and Farm 3 (beginning of fattening: Farm 1: 3.3 ± 1.0 s vs. Farm 3 3.6 ± 1.1 s; *p* < 0.05; middle of fattening: Farm 1: 3.6 ± 1.0 s vs. Farm 3: 3.5 ± 1.1 s; *p* < 0.05; end of fattening: Farm 1: 4.3 ± 1.0 s vs. Farm 3: 3.1 ± 1.1 s; *p* < 0.05). There was a significantly lower NC on Farm 2 than on Farm 3 at the beginning of fattening (Farm 2: 1.9 ± 1.0 s vs. Farm 3: 3.6 ± 1.1 s; *p* < 0.05) and no significant differences between the NC of Farm 2 and Farm 3 at the middle and end of fattening (middle of fattening: Farm 2: 2.8 ± 1.0 s vs. Farm 3: 3.5 ± 1.1s; *p* > 0.05; end of fattening: Farm 2: 2.5 ± 1.0 s vs. Farm 3: 3.1 ± 1.1 s; *p* > 0.05).

##### Differences Between the Points of Testing Within Each Farm

On Farm 1 and Farm 2, there was a significant increase in NC from the beginning to the end of fattening (Farm 1: 3.3 ± 1.0 s vs. 4.3 ± 1.0 s; *p* < 0.05; Farm 2: 1.9 ± 1.0 s vs. 2.5 ± 1.0 s; *p* < 0.05). No significant differences were observed between the NC at the beginning and end of fattening on Farm 3 (3.6 ± 1.1 s vs. 3.1 ± 1.1 s; *p* > 0.05).

#### 3.2.4. Gender NOT

The female pigs showed significantly lower AL and higher DC than the castrated male pigs (Table 1). There were no significant differences observed between the NC of the female and castrated male pigs (Table 1).

### 3.3. Relationship Between the Behavioral Variables 

In the HAT, the AL was negatively correlated with the NC and DC (Pearson correlation coefficient (r_p_ = −0.62, *p* < 0.001; r_p_ = −0.68, *p* < 0.001). The NC was positively correlated with the DC (r_p_ = 0.76, *p* < 0.001) (Table 2).

In the NOT, there was a positive correlation observed between the NC and DC (r_p_ = 0.74, *p* < 0.001) and there were negative correlations between the AL and the NC respectively DC (r_p_ = –0.55, *p* < 0.001 respectively r_p_ = −0.61, *p* < 0.001) (Table 2).

The DC of the NOT was positively correlated with the NC respectively DC of the HAT (r_p_ = 0.21, *p* < 0.001 respectively r_p_ = 0.24, *p* < 0.001) (Table 2).

## 4. Discussion

The present study aimed to examine whether behavioral tests like the HAT or NOT performed with fattening pigs of two different housing systems are useful to assess their positive affective state.

In the following, the behavioral variables AL, DC, and NC are discussed together for both behavioral tests. The AL indicated definite differences in the pigs’ behavior between the two different housing systems in both the HAT and NOT, especially at the middle and end of fattening. The pigs housed in the barren environment showed lower AL than the enriched-housed pigs. The same pattern of behavior was observed for the DC in the HAT, but not in the NOT. Finally, the NC showed no definite differences of the pigs’ behavior between the two housing systems, neither in the HAT nor in the NOT.

### 4.1. Approach Latency (AL)

According to Brown et al. [18] quicker latencies to approach novel stimuli, such as unknown humans or novel objects, are associated with less fearful animals. Therefore, it could be imaginable that these barren-housed pigs, which showed significantly quicker AL, possess a more positive affective state due to less fearfulness, but the quicker AL could also be a reason for a stronger motivation to explore novel stimuli [19] related to boredom in the environment of these barren-housed pigs. Additionally, Wemelsfelder et al. [20] argued that enriched-housed pigs might be less fearful or more curious to explore a novel object or a person in the home pen. But, simultaneously, barren-housed pigs seemed to be more motivated to explore a novel object or a person in the home pen probably due to fewer exploration possibilities in their habitat compared to enriched-housed pigs. Further, Forkman et al. [1] claimed that the avoidance reaction of the animal appears to be essential as both a non-curious and a fearful animal will show long latencies to approach. Consequently, it is difficult to determine whether the quicker AL of the barren-housed pigs might represent behavioral indicators for a more positive affective state possibly due to less fearfulness since other authors maintain that barren environments have negative effects on pig welfare [21]. Thus, it could be also conceivable that these quicker AL of the pigs housed in the barren habitat describe their more negative affective state, possibly due to boredom and fewer exploration opportunities in their environment.

The decreasing AL from the first to the third points of testing during fattening on Farm 1 and Farm 2 both in the HAT and NOT could be related to a gradual reduction in fear; Wemelsfelder et al. (2000) also observed a decreasing latency to enter the test arena for all pigs while the experiment progressed. Additionally, Forkman et al. [1] suggested that fear and anxiety decrease with age, which could also explain the decreasing AL during fattening on Farm 1 and Farm 2. The reduced level of fear and anxiety probably also causes the pigs to get used to unknown humans or novel objects [1] so that AL decreases when fattening progresses.

Moreover, an intensive monitoring of the avoidance reaction of the pigs (e.g., regarding a high latency to approach the unknown human or the novel object), might be able to evaluate whether the pigs show avoidance reactions or do not approach the novel stimuli due to a lack of interest (possibly related to various exploration opportunities in the environment) or fear and anxiety accompanied by flight behavior. The implementation of a forced human approach test might be a possible solution to detect the reason for the avoidance reaction of the pigs. Animals which flee when humans approach them are known to be fearful [22]. Waiblinger et al. [23] suggested that forced and voluntary approach tests measure the animals’ behavior in differing ways: the forced approach test might increase the likelihood of an animal responding more actively to the human. In the voluntary approach test, the chances of getting no response or a passive response might probably be higher. Hence, related observations between high approach latencies and the avoidance reaction could provide more detailed information about a more positive, respectively more negative affective state of the examined pigs.

### 4.2. Duration of Contacts (DC) 

In the HAT, there was a longer DC observed in the barren housing system (Farm 1) than in the enriched housing system (Farm 2 and Farm 3) at the middle and end of fattening. This definite difference between the DC of the pigs in the two different housing systems could also be related to the level of motivation to explore novel stimuli. Stolba and Woodgush [24] argued that the pigs’ interaction with a novel object decreased with increasing environmental complexity and that the pigs in more intensive housing systems were more interested in the novel object and that their interest was maintained for a longer period. Consequently, this longer DC related to a high level of motivation to explore novel stimuli in the barren-housed pigs could imply a behavioral indicator of less welfare and, therefore, a less positive affective state of these animals. Nevertheless, this definite difference in the pigs’ behavior between the two housing systems was only observed in the HAT. In the NOT, there were significant differences in the pigs’ behavior between the three farms, but not between the two housing systems. Hence, this might imply that the HAT and NOT do not measure the same behavioral attributes. Also, Boivin et al. [25] found no relationship between open-field tests and handling tests, possibly indicating that they do not reflect the same animal characteristic, which was also displayed in the weak correlations between the behavioral variables of the HAT and NOT. For example, in contrast to the NOT, the pigs’ behavior in the HAT was influenced by their previous experiences with humans. There are studies which demonstrate that negative handling leads to more avoidance responses [3].

Increasing DC was observed on Farm 1 and Farm 2 from the first to the third points of testing during the fattening both in the HAT and NOT. Regarding this, Forkman et al. [1] posited that human and object investigation increases with age, suggesting that fear and anxiety decrease with age and that there could be a habituation process that reduces the fear responses.

### 4.3. Number of Contacts (NC)

At the second point of testing in the HAT and third point of testing in the NOT, higher NC was observed in the barren housing system (Farm 1) than in the enriched housing system (Farm 2 and Farm 3). Reimert et al. [26] also observed barren-housed pigs more frequently present near the person than enriched-housed pigs. This can also be justified by less fearful pigs respectively a higher motivation for exploring novel stimuli but again it is not clear whether this pattern of behavior is related to a more positive or more negative affective state. This definite difference in the pigs’ behavior was only shown at one of the six points of testing during fattening in the HAT, respectively NOT.

The increased NC from the first to the third point of testing during fattening on farm 1 and farm 2 in the HAT and NOT might also be related to a habituation process [1], which lowered the level of fear and increased the NC of the pigs with the unknown human or the novel object.

### 4.4. Gender

The female pigs showed quicker AL and longer DC than the male pigs in both the HAT and NOT. This might indicate that female pigs are less fearful than male pigs. Reimert et al. [26] observed gilts approaching and touching a person and a novel object faster than the barrows suggesting the female animals were less fearful than the male ones. The gilts of this study also had lower basal cortisol concentrations than barrows [26], which could explain the reaction of the female pigs as less fearful since high cortisol concentrations depicted an indication of stress [27], and stress is known to be often connected with fear [1].

Further, especially the higher AL of the male pigs than the female pigs in the HAT can be explained by their castration and its consequences, since in studies by Reimert et al. [28] the castrated male piglets also responded more fearfully to novel situations than female piglets. Additionally, in this study, the male pigs had higher salivary cortisol concentrations than the female pigs [28], which could also indicate a stressed and fearful affective state, as mentioned before. It is supposed that the absence of gonadal testosterone makes the castrated pigs more fearful [28] and that handling by a human during castration and the connected pain afterwards makes the castrated male pigs more fearful of humans [29], so that they do not approach them as quickly as the female pigs do. Further studies with uncastrated boars would be useful to support these assumptions.

Additionally, the development of the brain could influence the exploratory behavior of animals; Fleming and Dilger [30] claimed that, in general, the female brain of pigs develops faster than the male ones possibly explaining the shorter AL and longer DC of the female pigs in both behavioral tests due to better developed cognitive abilities.

## 5. Conclusions

The results of the present study showed that the HAT and NOT performed with fattening pigs of two different housing systems might be suitable to assess their level of anxiety and fear. Simultaneously, the pigs also appeared to be capable of showing a stronger or lower motivation to explore novel stimuli making it difficult to draw a clear conclusion as to whether a high or low level of motivation to explore indicates more negative or more positive affective states. Conclusively, in this study, neither the HAT nor the NOT depicted autonomous, reliable indicators of positive affective states in growing pigs. However, the results of this study lay essential foundations for further investigations concerning the assessment of the positive affective state of fattening pigs. Furthermore, the findings of this study provide more detailed information about certain reactions to unknown humans or novel objects of fattening pigs which are housed in different environments.

## Figures and Tables

**Table 1:** Least squares means and standard errors (s) (retransformed) of the behavioral variables in the HAT and NOT of different genders.

Behavioral Variables	Female	Male	*p*-Value
HAT-AL	33.36 ± 1.09 ^a^	52.05 ± 1.11 ^b^	0.04
HAT-DC	10.97 ± 1.10 ^a^	7.75 ± 1.12 ^b^	0.005
HAT-NC	1.96 ± 1.03 ^a^	1.82 ± 1.03 ^a^	0.05
NOT-AL	12.52 ± 1.08 ^a^	16.14 ± 1.09 ^b^	0.01
NOT-DC	25.98 ± 1.08 ^a^	20.95 ± 1.10 ^b^	0.03
NOT-NC	3.23 ± 1.03 ^a^	3.04 ± 1.03 ^a^	0.1

HAT = human approach test; NOT = novel object test; AL = approach latency; DC = duration of contact; NC = number of contacts; ^a, b^ different letters indicate significant differences between the gender (*p* < 0.05).

**Table 2:** Pearson rank correlations between the residuals of the linear mixed models of the behavioral variables.

Behavioral Variables	HAT-DC	HAT-AL	NOT-NC	NOT-DC	NOT-AL
HAT-NC	**0.76**	**−0.62**	0.16	**0.21**	−0.16
HAT-DC		**−0.68**	0.16	**0.24**	−0.19
HAT-AL			−0.13	−0.18	0.18
NOT-NC				**0.74**	**−0.55**
NOT-DC					**−0.61**
NOT-AL					

Correlation coefficients in bold indicate moderate respectively high and statistically significant correlations (*p* < 0.05).
